# Severe Congenital Pulmonary Valve Stenosis Diagnosed in Adulthood: A Case Report

**DOI:** 10.7759/cureus.103630

**Published:** 2026-02-14

**Authors:** Mehdi Moujahid, Hafsa Erregui, Najat Mouine, Zouhair Lakhal, Aatif Benyass

**Affiliations:** 1 Cardiology, Military Hospital Mohamed V, Rabat, MAR

**Keywords:** adult congenital heart disease, balloon pulmonary valvuloplasty, pulmonary valve stenosis, resource-limited settings, transthoracic echocardiography

## Abstract

Pulmonary valve stenosis is a congenital heart disease that is usually diagnosed during childhood, while presentation in adulthood is uncommon and may lead to delayed diagnosis. We report the case of a 21-year-old patient with no significant medical history who presented with a six-month history of exertional dyspnea and constrictive chest pain. Electrocardiography showed right axis deviation with signs of right ventricular hypertrophy, and chest radiography revealed dilatation of the left pulmonary artery. Transthoracic echocardiography demonstrated severe valvular pulmonary stenosis with a peak velocity of 4.53 m/s and a maximum systolic gradient of 82.23 mmHg, associated with right ventricular hypertrophy and preserved systolic function, without evidence of pulmonary hypertension. Computed tomography pulmonary angiography confirmed focal post-stenotic dilatation of the left pulmonary artery. After a multidisciplinary discussion, balloon pulmonary valvuloplasty was recommended. This case highlights the importance of considering congenital pulmonary valve stenosis in young adults presenting with exertional symptoms and underscores the challenges of access to advanced interventional therapies in resource-limited settings.

## Introduction

Congenital pulmonary valve stenosis is a well-recognized congenital heart disease most commonly diagnosed during infancy or childhood, as significant obstruction usually leads to early clinical manifestations. However, mild or moderate forms may remain asymptomatic for years, allowing the condition to go unrecognized until adulthood [1,2].

Congenital heart disease affects approximately 1% of live births, and advances in pediatric cardiology and cardiac surgery have resulted in a growing population of adults living with congenital heart disease (ACHD) [3]. In this population, some congenital lesions, including pulmonary valve stenosis, may present later in life or remain undiagnosed until adulthood.

In adults, pulmonary valve stenosis may be discovered during evaluation for exertional dyspnea, chest pain, or abnormal cardiac findings. Delayed diagnosis can result in progressive right ventricular pressure overload, leading to right ventricular hypertrophy, reduced exercise tolerance, and post-stenotic dilatation of the pulmonary artery. Transthoracic echocardiography (TTE) remains the cornerstone of diagnosis and follow-up, enabling assessment of stenosis severity, right ventricular remodeling, and associated cardiac abnormalities [1-3].

We report a case of severe congenital valvular pulmonary stenosis diagnosed in a young adult, highlighting the diagnostic features, imaging findings, and therapeutic considerations, as well as challenges related to access to interventional treatment in resource-limited settings.

## Case presentation

A 21-year-old male patient with no significant past medical history presented with a six-month history of exertional dyspnea (New York Heart Association class II) associated with constrictive chest pain on exertion, occurring even with minimal physical activity. There was no history of syncope, palpitations, or cardiovascular risk factors.

**Figure 1:**
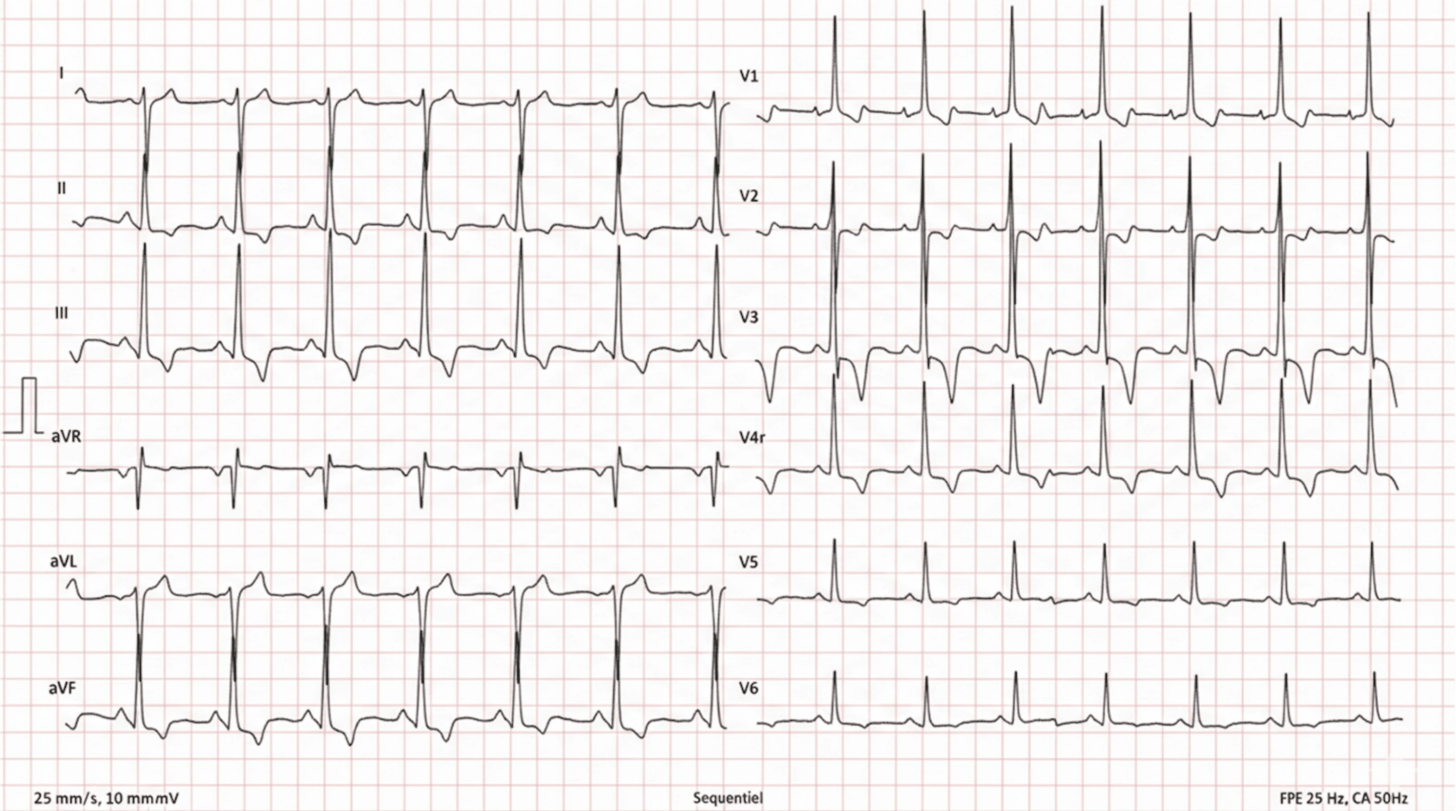
Electrocardiographic Findings in Severe Pulmonary Valve Stenosis Twelve-lead electrocardiogram showing right axis deviation and features consistent with right ventricular hypertrophy, including tall R waves in the right precordial leads and deep S waves in the lateral leads. These findings reflect chronic right ventricular pressure overload secondary to severe pulmonary valve stenosis.

On physical examination, the patient was hemodynamically stable, with a blood pressure of 120/70 mmHg, heart rate of 78 beats per minute, and oxygen saturation of 98% on room air. There was no cyanosis and no digital clubbing. Jugular venous pressure was normal. Cardiac auscultation revealed a systolic ejection murmur best heard at the left upper sternal border. Lung auscultation was normal, and no peripheral edema was noted. Routine laboratory tests were within normal limits, including complete blood count, renal function, and electrolytes. Cardiac biomarkers, including troponin and B-type natriuretic peptide (BNP), were not elevated. Electrocardiography demonstrated right axis deviation with features consistent with right ventricular hypertrophy (Figure 1).

**Figure 2:**
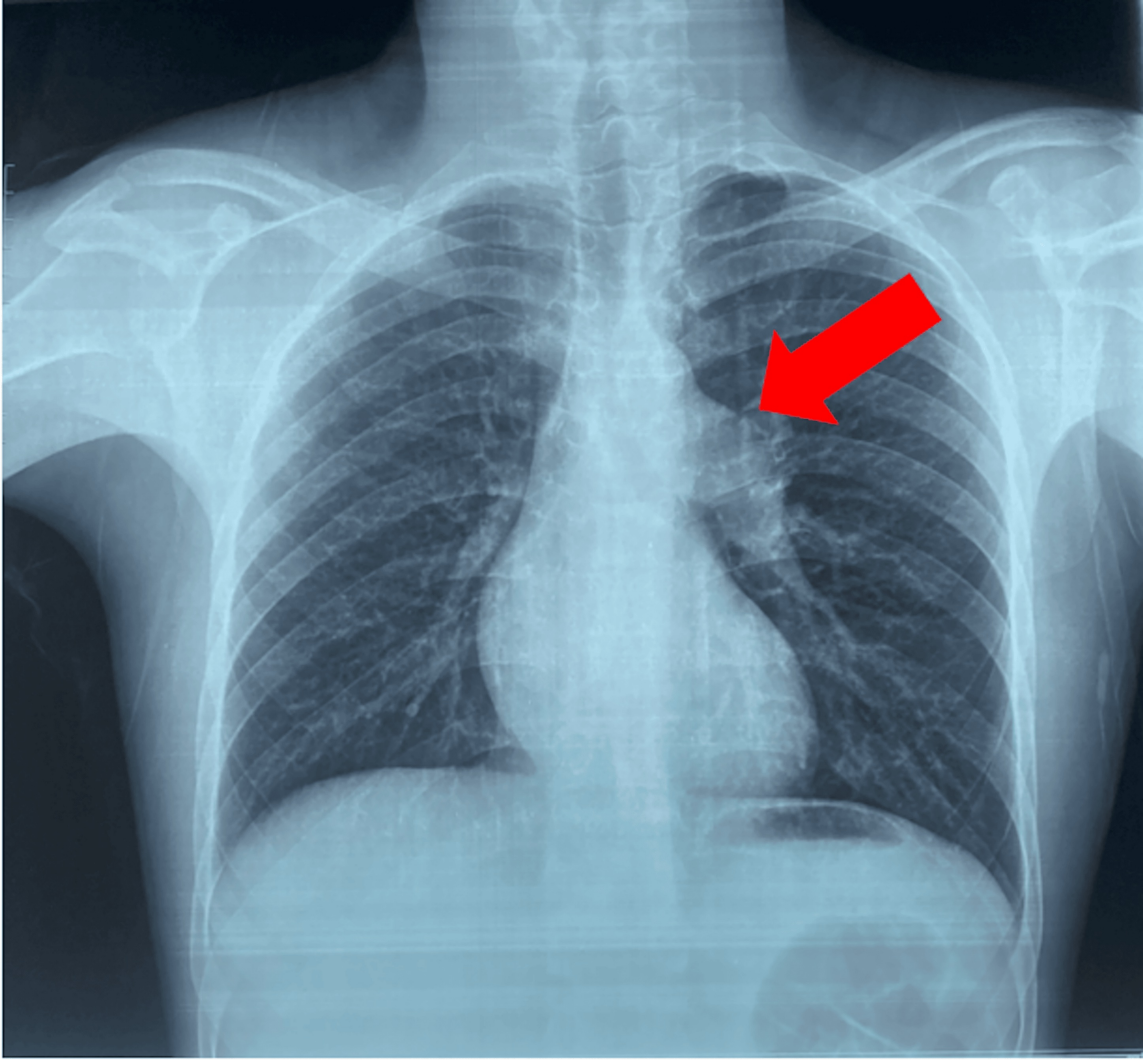
Posteroanterior Chest Radiograph Showing Left Pulmonary Artery Dilatation A prominent left pulmonary artery segment (red arrow) is seen, consistent with post-stenotic pulmonary artery dilatation in the setting of severe pulmonary valve stenosis. No signs of pulmonary congestion are observed.

Chest radiography showed a prominent left pulmonary artery segment without pulmonary congestion (Figure 2).

**Figure 3:**
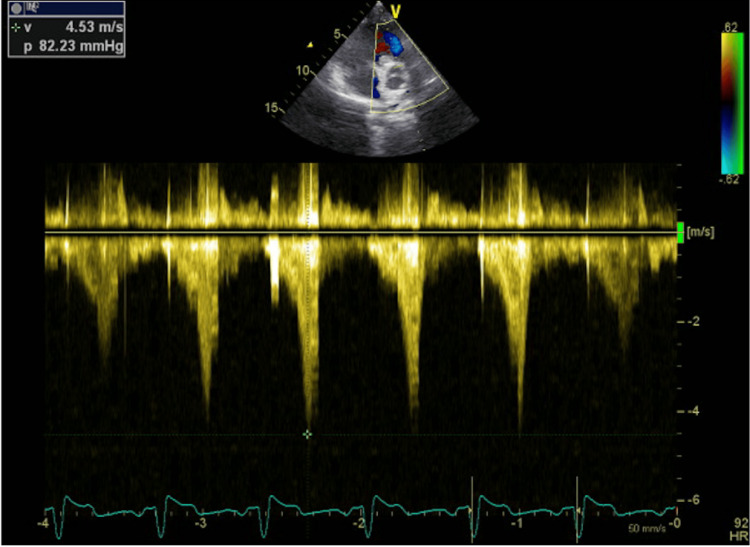
Continuous-Wave Doppler Echocardiography Demonstrating Severe Pulmonary Valve Stenosis. A markedly increased transvalvular flow velocity is seen, with a peak velocity of 4.53 m/s and a maximum systolic gradient of 82.23 mmHg, consistent with severe pulmonary valve stenosis. The Doppler profile reflects significant right ventricular outflow tract obstruction.

**Figure 4:**
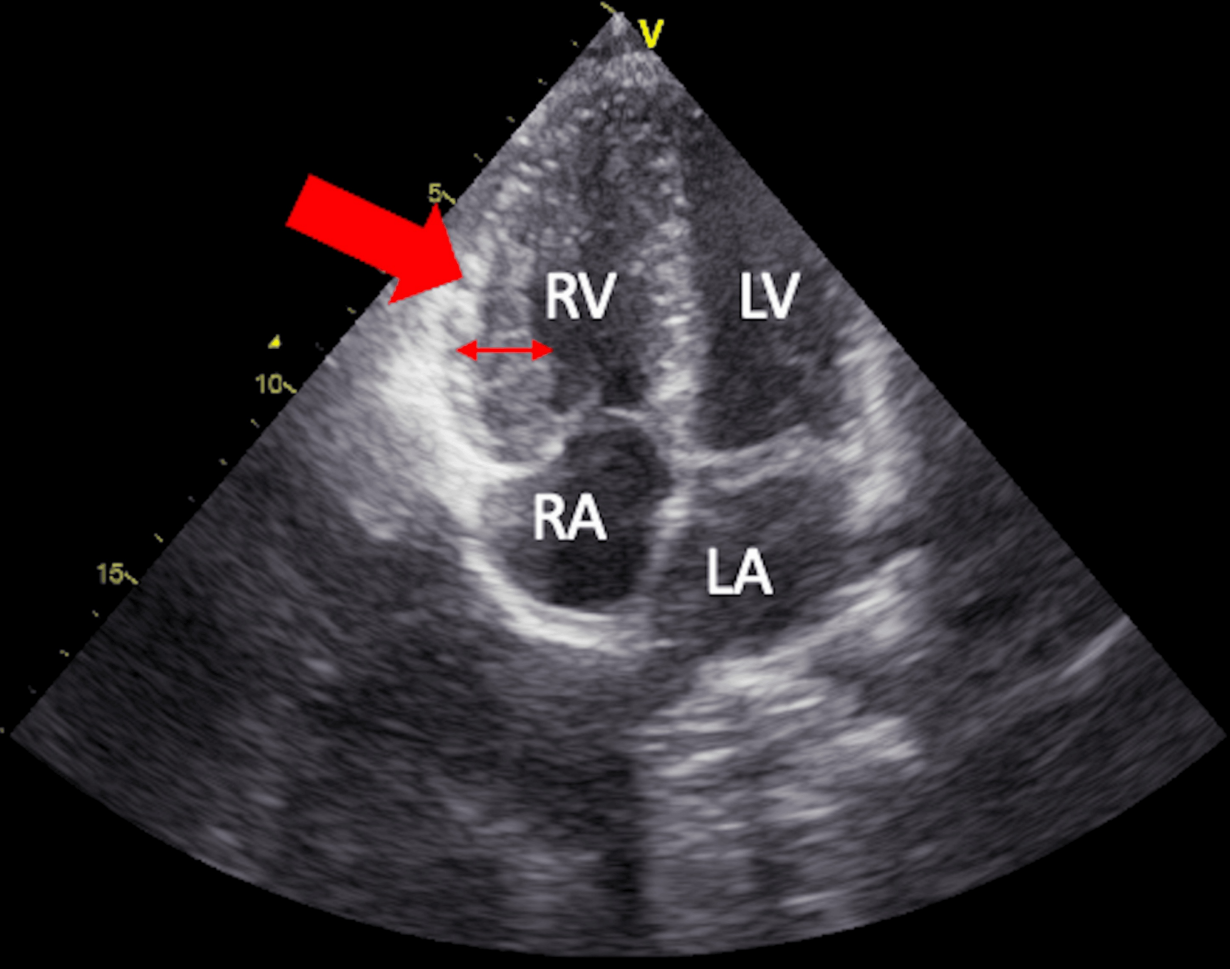
Apical Four-Chamber TTE Showing Right Ventricular Hypertrophy Right ventricular hypertrophy (red arrow) is seen with preserved cavity size. The left-sided cardiac chambers appear normal. These findings reflect chronic right ventricular pressure overload secondary to severe pulmonary valve stenosis. RV: right ventricle; LV: left ventricle; RA: right atrium; LA: left atrium; TTE: transthoracic echocardiography

TTE revealed severe valvular pulmonary stenosis with a peak velocity of 4.53 m/s and a maximum systolic gradient of 82.23 mmHg across the pulmonary valve (Figure 3). The right ventricle was hypertrophied with preserved systolic function (Figure 4). No other significant valvular abnormalities were identified, and there were no echocardiographic findings suggestive of pulmonary hypertension.

**Figure 5:**
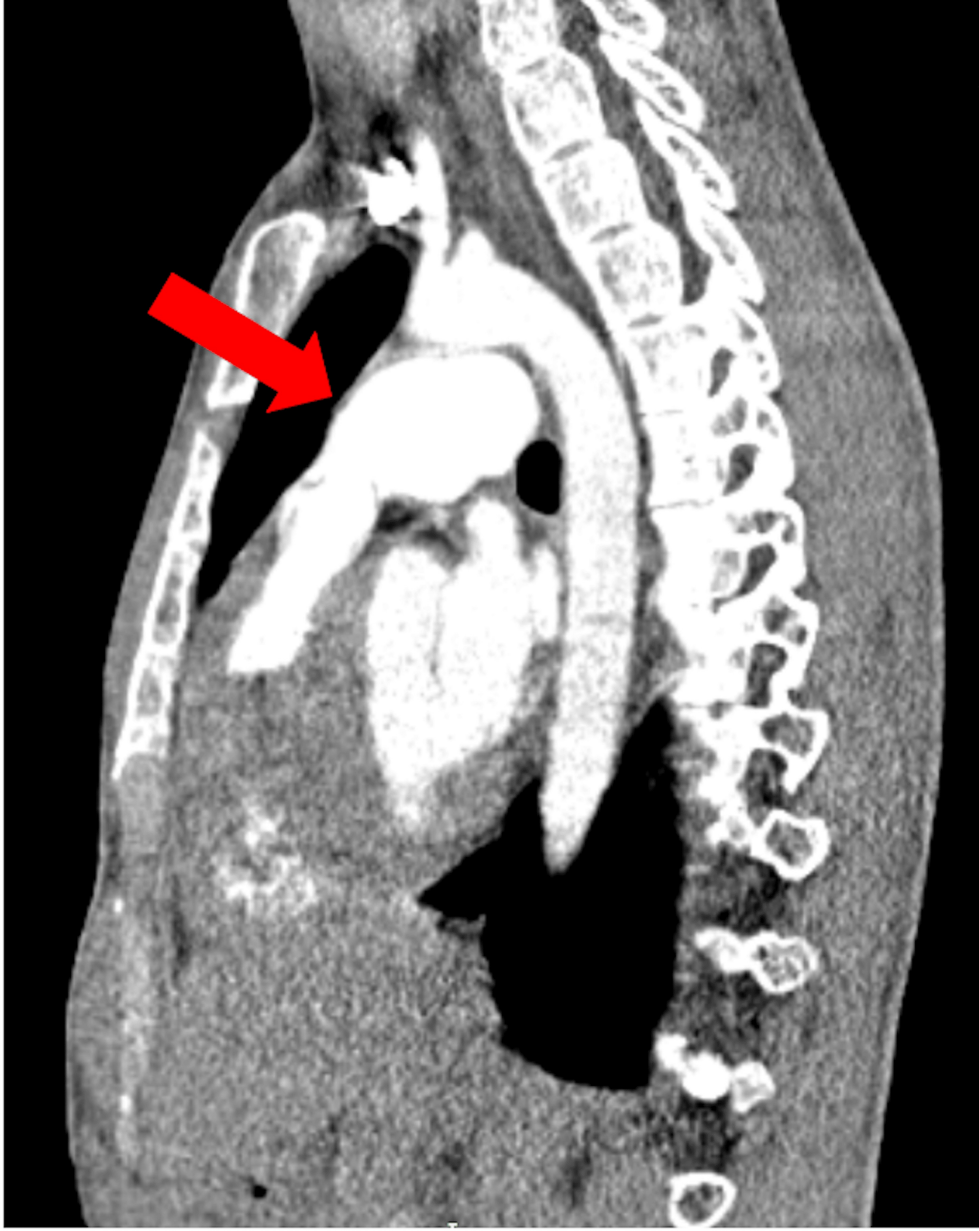
Sagittal CTPA Showing Post-Stenotic Dilatation of the Left Pulmonary Artery Marked dilatation of the left pulmonary artery (red arrow) is seen, consistent with post-stenotic pulmonary artery dilatation secondary to severe pulmonary valve stenosis. No evidence of pulmonary embolism or associated aortic coarctation is observed. CRPA: computed tomography pulmonary angiography

**Figure 6:**
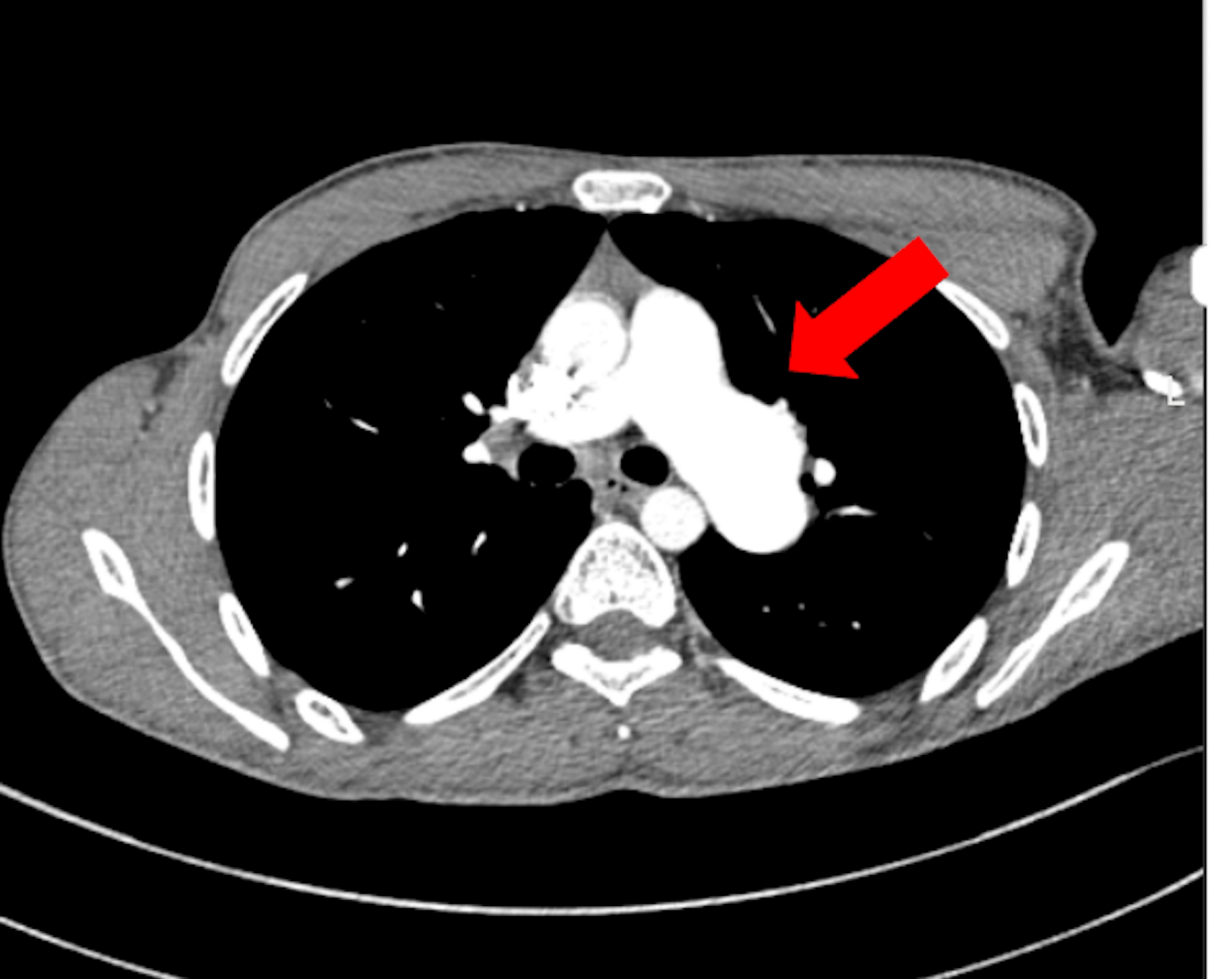
Axial CTPA Showing Left Pulmonary Artery Dilatation Marked dilatation of the left pulmonary artery (red arrow) is seen, consistent with post-stenotic pulmonary artery dilatation in the setting of severe pulmonary valve stenosis. No intraluminal filling defects suggestive of pulmonary embolism are observed. CTPA: computed tomography pulmonary angiography

Computed tomography pulmonary angiography (CTPA) demonstrated focal dilatation of the left pulmonary artery without evidence of pulmonary embolism, supporting post-stenotic pulmonary artery dilatation (Figures 5, 6). Differential diagnoses such as pulmonary embolism, subvalvular obstruction, and other causes of right ventricular outflow tract obstruction were considered and excluded based on echocardiographic and computed tomography findings

Following a multidisciplinary medico-surgical discussion, balloon pulmonary valvuloplasty was recommended. However, as this procedure was not available at our institution, the patient was referred to a center abroad where the intervention was performed. At the time of writing the report, post-intervention outcomes were not yet available.

## Discussion

Pulmonary valve stenosis is most commonly congenital and typically results from commissural fusion, leaflet thickening, or valve dysplasia. The severity of obstruction determines the timing of presentation. While severe forms usually manifest early in life, delayed presentation in adulthood may occur in patients with slowly progressive disease or good right ventricular adaptation [1,2]. In adults, congenital heart disease may remain asymptomatic for many years or be incidentally detected, with clinical presentation largely depending on the severity of the underlying lesion [3].

In adults, symptoms such as exertional dyspnea, chest pain, or syncope reflect the limited ability of the pressure-loaded right ventricle to increase cardiac output during exertion. Electrocardiographic findings often include right axis deviation and signs of right ventricular hypertrophy, as observed in our patient. Chest radiography may show post-stenotic dilatation of the pulmonary artery, a supportive feature in the appropriate clinical context [2].

TTE is the first-line imaging modality for diagnosis and severity assessment. Current guideline-based thresholds classify pulmonary valve stenosis as severe when the peak transvalvular velocity exceeds 4.0 m/s or the peak systolic gradient is greater than 50 mmHg. In our case, the measured peak velocity of 4.53 m/s and gradient of 82.23 mmHg clearly indicated severe stenosis, associated with right ventricular hypertrophy but preserved systolic function and no evidence of pulmonary hypertension. CTPA supported the diagnosis by demonstrating focal post-stenotic dilatation of the left pulmonary artery and excluding pulmonary embolism [3,4].

Management of severe pulmonary valve stenosis depends on symptom status, stenosis severity, and valve morphology. Percutaneous balloon pulmonary valvuloplasty is considered the preferred treatment for symptomatic patients with severe valvular stenosis and favorable anatomy because of high success rates and acceptable safety. According to current European Society of Cardiology (ESC) guidelines for adult congenital heart disease, balloon pulmonary valvuloplasty is recommended as first-line therapy for symptomatic severe valvular pulmonary stenosis (Class I recommendation), with procedural success rates exceeding 90% in suitable valve anatomy. Surgical intervention is generally reserved for patients with dysplastic valves, associated subvalvular or supravalvular obstruction, or when percutaneous treatment is not feasible [2,3,5,6].

In the present case, a multidisciplinary heart team recommended balloon pulmonary valvuloplasty. However, the lack of local availability of this technique required referral abroad, illustrating real-world barriers to guideline-directed care in resource-limited settings. At the time of publication, post-procedural outcomes were not available.

This case emphasizes the importance of considering congenital heart disease in young adults with exertional symptoms and highlights the central role of echocardiography in diagnosis and management planning. Early recognition and timely intervention are essential to prevent long-term right ventricular dysfunction and improve functional status.

## Conclusions

Severe congenital pulmonary valve stenosis may remain clinically silent until adulthood and should be considered in young adults presenting with exertional symptoms. TTE plays a pivotal role in establishing the diagnosis, assessing severity, and guiding management. Balloon pulmonary valvuloplasty is the treatment of choice for symptomatic severe valvular stenosis when feasible. This case highlights the importance of early recognition and the challenges of access to advanced interventional therapies in resource-limited settings.
